# SYMPOSIUM ON OPTICAL FIBER MEASUREMENTS

**DOI:** 10.6028/jres.092.007

**Published:** 1987-02-01

**Authors:** G. W. Day, D. L. Franzen

**Affiliations:** Program Chairman and General Chairman, respectively for the Symposium, Electromagnetic Technology Division, National Bureau of Standards, Boulder, CO 80303

One might expect that, as a technology matures, associated measurement problems would be solved and research in those areas would diminish. That view is supported by examination of the programs of some of the major international conferences related to optical fiber communications, which have shown a steady decline in the percentage of papers related to measurements. It is not supported by experience with the biennial Symposium on Optical Fiber Measurements. Rather, it appears that the need for such a specialized meeting on the topic of measurements continues to grow.

The 4th Symposium, sponsored by NBS in cooperation with the Optical Society of America and the IEEE Optical Communications Committee was held September 9–10, 1986, at the NBS Boulder Laboratories. It drew nearly 350 people from 17 countries to hear 29 contributed and 5 invited papers. The papers of this years’ program were exceptionally diverse, both in subject and origin. In only two technical areas, chromatic dispersion and mode-field diameter measurements, was it possible to schedule a full session on a single topic. In origin, there were 21 organizations in 9 countries represented in the program. Over 40 percent of the papers were from outside the United States.

One reason for the continued interest in fiber measurements, suggested by W. T. Anderson of Bell Communications Research in an invited paper that opened the Symposium, is that while the properties of silica fibers are near their fundamental limits, more complete exploitation of their capacity continues to add to the characterization requirements.

Continuing concern with chromatic dispersion measurements arises from a growing interest in designing single-mode fiber communications systems for very high data rate operation. The limitation imposed by the fiber in these systems is a combination of material dispersion and waveguide dispersion, which cause different spectral components from the source to propagate at slightly different velocities. Material dispersion, which is related to the second derivative of the refractive index with wavelength, is fairly predictable, passing through zero at a wavelength near 1.3 μm. Waveguide dispersion, which depends on the shape of the refractive index profile, adds to the material dispersion, shifting the wavelength of zero total dispersion (i.e., the optimum operating wavelength) accordingly.

System designers need to know both the wave-length, λ_0_ of zero total dispersion, and the variation of the dispersion around that wavelength. The latter requires a satisfactory model. A paper by Reed and Philen of AT&T Bell Laboratories concluded that a three term Sellmeier dispersion relation, well known in optics, is an adequate model for the most common types of fiber. For so-called dispersion shifted fibers, designed to have λ_0_ near the wavelength of minimum loss at 1.55 μm, however, they found that it was necessary to use a more complex form of the Sellmeier equation.

Early dispersion measurement techniques required elaborate and expensive equipment, hence a strong interest in simpler measurements. A paper from Phillips Glass reports very encouraging results with a method called the phase-shift technique. In this technique the phase shift of a modulated source is monitored as its wavelength is scanned. The authors conclude that an accuracy of better than 0.5 ps/nm · km can be achieved but to do so will require a temperature stabilization of the fiber to better than 1 K during the measurement.

Because the losses of modern fibers are extremely low, a much greater concern is attached to losses incurred in splices. One aspect of this problem is the need to establish and test high quality splices in the field. Techniques for field testing were reviewed in an invited paper by Reinhard Engle of Siemens and Everett McNair of Siecor. Another aspect is adequate specification of the mode profiles of single mode fiber. Several contributed papers seemed to conclude that it is relatively easy to achieve measurement consistency on step index single mode fibers but that more advanced index profile designs still pose problems.

Three invited talks discussed programs of related and future interest to fiber measurement specialists. These included a discussion of source and detector characterization by Ito and Kurumada of NTT, a summary of the characterization of advanced fibers and devices by Payne, et al., of the University of Southampton and British Aerospace, and a discussion of the problems associated with characterizing planar optical waveguides by Alferness of AT&T Bell Laboratories.

The Technical Digest for the Symposium on Optical Fiber Measurements, 1986, contains summaries of all invited and contributed papers. It is available as NBS Special Publication 720 from the Superintendent of Documents, U.S. Government Printing Office, Washington, D.C. 20402 ($8.00).

